# First District Dental Society of New York

**Published:** 1887-03

**Authors:** 


### FIRST DISTRICT DENTAL SOCIETY.

REPORTED EXPRESSLY FOR THE INDEPENDENT PRACTITIONER.

The First District Dental Society of New York celebrated its eighteenth anniversary on Monday, Tuesday and Wednesday, January 17th, 18th and 19th. The programme was very long, the whole beginning with a meeting on Monday evening, January 17th, at 8 o'clock, which was held in the Grand Lodge room of the Masonic Temple, corner of 23d Street and 6th Avenue. The attendance at the meeting was estimated to be over 500, dentists coming from almost every State in the Union. It was opened with prayer by Brady E. Backus, D. D., of New York, followed by an adddress of welcome by the President, Dr. Wm. Carr, who, in the course of his remarks, spoke of the International Medical Congress as follows:

At a special meeting of our Society, held on November 15th, a paper, entitled "Dentistry Not a Specialty in Medicine," was read by Dr. Norman W. Kingsley, in which he advocated holding an International Dental Congress, to which all reputable dentists should be entitled to membership. While this meets the approval of many of us, we feel that it is as yet a question for future consideration, as one of the most important events, not only to us as a Society but to the dental profession generally, will occur during the present year, on September 5th, when the International Medical Congress will convene at Washington. While the excellence of American dentistry is universally conceded, we have never had the opportunity of meeting our foreign brethren of different nationalities, as a professional body, in our own land. This opportunity will be offered at the coming session of the Congress, to which a section has been assigned in Oral and Dental Surgery. In regard to this section, there has existed on the part of some of us a feeling of apathy for which no one seems able to furnish an explanation. The section was not organized for the aggrandizement of any individual or class of individuals, but it was organized that dentistry of the civilized world might be properly represented.

It is our duty to cast aside all personal and sectional differences and zealously assist the officers of this section to make it worthy, as a scientific body, of a place in the Congress, and thus prove that the reputation that has been so universally accorded us is not unmerited. We must also consider that we have a reputation for hospitality to sustain, and we should receive our foreign brethren with a generous and hearty welcome.

It is for the dentists of America to decide whether this section shall be a success or a failure. If it proves to be a success, we can view the result with pardonable pride ; if it proves to be a failure, the disgrace will rest upon us as a profession.

I beg you, gentlemen, to do whatever lies in your power to make this section worthy of Americans and American dentistry.

Prof. Taft, of Ohio, responded, in substance, as follows :

*Mr. President and Gentlemen :*

To the words of hearty and inspiring welcome to which we have just listened, what shall be said befitting the occasion, in response ? I have no doubt that the rich provision that has been made for this meeting will be a surprise, and afford much gratification to the visitors and guests here assembled. No scheme or programme equal to this in kind or extent has ever been presented for a similar occasion, and it may fairly be accepted as one of the marked indications of our professional progress ; and it is eminently fit and proper that it should be done in this great city, to which so much of history attaches, where scenes have been enacted and men lived that will be ever held in remembrance. It was here that, one hundred and twenty years ago, Robert Wolfingdale lived, the first dentist who plied his vocation on American soil. Here also lived John Greenwood, so successful in practice. This, too, was the home of Elisha Baker, John Lovejoy, I. N. Foster, J. Smith Dodge, Solyman Brown, Shearjashub Spooner, E. G. Tucker, Eleazer Parmly, and a host of worthies who accomplished a great work, and who wrought better than they conceived by laying the foundation on which is now being reared a noble structure. Here was published the first periodical in the world devoted to the interests of dental science and art. Here the American Society of Dental Surgeons, the first dental society in the world, was organized, and by noble men, whose aims and aspirations it would be well to imitate ; whose lives and works it would be well to study. Though this organization lasted but a few years, much good work was done, and the spirit it engendered has ever been active, and will never be extinct. It did the work of a pioneer, and laid out a path for others to travel. That its aim was high and praiseworthy is demonstrated by the first article of its constitution : "The objects of this Society are to promote union and harmony among all respectable and well-informed Dental Surgeons ; to advance the science by free communication and interchange of sentiments, either written or spoken, between members of the Society, both in this and other countries ; in fine, to give character and respectability to the profession by establishing a line of distinction between the truly meritorious and skillful, and such as riot in ill-gotten fruits of unblushing impudence and empiricism." Also from the pen of Dr. Solyman Brown, Secretary of the Society, written soon after its organization, in answer to this question : "What are the essential qualifications for membership in the American Society of Dental Surgeons ?" he states : "Respectable talents, creditable acquirements, professional integrity and good moral character." Who to-day can improve on this definition of the qualification of a good dentist ?

These noble men were deeply interested in all efforts for the advancement of dental science, and they and their followers did pioneer work all over the land. In this historic place it is eminently proper for us to gather, for here our profession saw its first light in the new world, and we should honor the memories of those men who made possible the achievements of the present day. We should well consider the work they have done for us by clearing away difficulties, that their followers might tread an easy path, and we should honor the memories of those who laid foundations on which we build, and sowed seeds that bear such fruits as we now enjoy.

Let us bear in mind that the pioneers are not all of yore, for some are even with us now. We have still fresh fields to occupy, and new avenues to open, with difficulties to be encountered and sacrifices to be made. Seldom does the pioneer receive the appreciation he merits.

We can look with pleasure on many things pertaining to our profession, but perhaps none for which we should be more thankful than for the solidity and absence of schism among us. There is no principle of discord to produce contending factions. Occasional agitations that have occurred have, after a brief period, passed away. Our body moves forward in solid column, to accomplish whatever great objects we desire to obtain, and while all of us are supposed to be independent thinkers, and capable of drawing our own conclusions whenever important questions affecting the whole body come up, there is such an unanimity of views and concurrence of action that no parallel can be found. This is no vain boast, for facts verify the statement. Nowhere can a body of men be found so free, yet so firmly banded together, without division or faction, and the truth of this statement is manifested on this present occasion. In this assembly are hundreds from all parts of this broad land, for a common purpose, with common sympathy and strong fraternal feelings. And thus do we occupy enviable ground, which should be utilized in discharging the duties and responsibilities devolving upon us. We have no intestinal struggles in which to spend our strength or divert our energies, and naught to do but go on and fill the high behest before us. Is it then a marvel that such great and rapid progress should be made, and that in the race for high achievements dentistry should be abreast of even the foremost? Let us be careful that our work does not lag. The Dental Profession is one of the battalions of the great army engaged in the warfare against disease and the ruin which it works. Let us then press on to that great victory, when disease shall be conquered and death shall no longer sit regnant as "king of terrors," but shall be relegated to the position of the kindly porter to open the gate for the easy passage of redeemed humanity from this to a higher place of activity and enjoyment.

The President then called upon Prof. James Truman, of Philadelphia, who read a paper on "The Rational Basis of Practice." The essayist remarked that his paper was not intended to bring up anything new, but to stir up thoughts. He remarked that principles should be our corner-stones in practice, and as our field was too large he advocated specialties in dentistry as the only way to success. The older men had not made as much comparative progress as the younger members of our profession. He admitted that our fathers in dentistry had been great men, deserving great credit, but they saw too much before them, and had no time for investigating small things. The essayist then described at some length the growth and development of an infant, and in this connection, while discussing the shedding of teeth, he highly recommended the use of the lancet at a proper time, whenever dentition was accompanied with pain and the gums swollen. The roots of the teeth at that time were not fully formed, and consequently their apices were sharp, and by the pressure of the gum upon the crown of the tooth they produced an irritation upon the dental pulp. He was opposed to the extraction of the first permanent molars in any case, and discussed at some length the comparison between the teeth of man and those of rodents.

The essayist then described the structure of the pericementum, as well as some of its pathological conditions, defining pericementitis and pyorrhœa alveolaris. In this connection he doubted if, in the pericementum of a tooth which had been out of the mouth for any length of time, the circulation could become re-established, although he acknowledged that the membrane was governed by laws different from those which dominate other tissues. He objected to the use of clamps, as well as separators, and was of the opinion that all continuous force applied to the pericementum for any length of time might result in death, either of the alveolus or the dental pulp. Some of the bad effects caused by the injudicious use of such instruments could only be observed from twenty-four to forty-eight hours after the operation. As a preventive of an inflammatory reaction in these conditions, the essayist advised the use of a germicidal mouth-wash. He condemned all immediate wedging, but recommended the use of cotton for such purposes. He was not in favor of placing bands around the roots of teeth, which encroach upon the pericementum, thereby causing either acute or chronic inflammation, while extensive bridge-work, which was supported by a few roots or teeth only, was open to the same objections. The essayist was opposed to the use of all bridge-work from that standpoint, as well as for the reason that it might become loose and be accidentally swallowed by the patient.

Dr. Truman then discussed several of the causes of caries. He is of opinion that, very often, the cervical decay at the margins of proximate fillings is caused by the use of the mallet. Wherever a mallet was employed for the introduction of gold into cavities of teeth, the operator should always avoid its touching the walls of the cavity. In many instances the enamel was cracked, and decay might commence through the invasion of micro-organisms. He indorsed the theories of Miller, and of Leber and Rottenstein, and believed that galvanic action had never been proven the cause of caries. He thoroughly believed in the Herbst method of burnishing gold against the walls of cavities, since he had become more familiar with the method.

He closed his paper by reminding dentists that the more they study the causes of decay, the better will be the results of their practice.

Dr. T. D. Shumway, of Plymouth, Mass., then read a paper, entitled "A Monograph of Science and Dentistry." The essayist remarked that all parts of the teeth were subject to decay, and advised a careful study of the causes which, as yet, were not definitely known to every practitioner. He then dwelt at some length upon restoring the contour in filling teeth, but was opposed to separations. He was opposed to making experiments in the filling of teeth out of the mouth, as very often two experimenters arrived at opposite conclusions in making the same experiment. He thought that the causes of dental caries had not been satisfactorily explained, and was in doubt whether the bacteria preceded the acid, or *vice versa*. He dwelt upon the necessity for the study of physiology before that of pathology, and described the development of the enamel and dentine in a very unique manner.

He expressed the belief that most forms of dental caries originated from predisposition, and that the causes were located more in the tooth substance itself than in its surroundings. The chemico-vital force was an energy which ought to be better considered. The filling of a tooth must be scientific as well as practical, and although gutta percha, and tin and gold as filling materials, had proved to be reliable, yet most operators did not pay sufficient attention to the physical properties of these materials. Gold, under certain conditions, was the best material, but when used against the walls of teeth it should not be annealed, as heat would develop a new crystallization of the gold, and this would prevent perfect adaptation to the walls of the cavity. He condemned the use of the mallet, and dwelt upon the idea of teaching only one method of filling, which had for its base the highest law of physical force.

Dr. Wm. H. Atkinson said that the human body arises out of a mucoid bleb of protoplasm—the germ. A stratum of hyper-oxidized hydrocarbon forms the outer skin of this little body, and an inner lining, which are the analogical foresteps of the respiratory, digestory and genito-urinary tracts. Between these two skins known as the "epi-blast" and "hypo-blast" masses of embryonal or indifferent corpuscles make their appearance at the margins of these membranes by proliferation, which are gathered into proto-vertebral and which subsequently differentiate under the incoming current of typal impact into nerve plates, bone plates and muscle plates, from which the nervous, bony and muscular systems take origin.

These masses are divided into body walls ("somato-pleural") and visceral walls ("splanchno-pleural").

There are two forms of limitory tissue, viz: Epithelial and white connective tissue corpuscles, which form the limits of the body and of the cavities of the viscera, and of the viscera and limbs, constituting the system of organs as a whole.

Nutrition occurs in the protoplasmic or neural mass constituting the interiors of the ultimate corpuscles of the various tissues, nervous, bony, muscular, connective and epithelial. The incoming current of pabulum nourishes these tissue elements in health. When the nutrient impact is obstructed irritation is produced, which arrests nutritional changes in the bodies (nucleus) of the corpuscles.

Should the obstruction be removed, nutrition goes on again without special detriment; but should the irritation be continued, combinations of pabulum with tissue cease, and reversal or dissociation of pabulum and corpuscles sets in.

This is the first and simplest form of retrogressive movement which constitutes the initial form of the imflammatory process.

Should this continue, the process of reversal of current is propagated to adjoining corpuscles, until the calibre of the trophic or afferent nerve is so encroached upon as to reduce the neurine, minifying this condition of the nutrient or sensory current.

Emotion or sensation may do this; words—unkindness may kill!

These are examples of shock.

After trophic and sensory nerves have been obstructed for a time, the interruption of current is propagated to vaso-motor nerves also, and then we have not only "stasis" of nerve current, but also stasis of blood current in the fine capillaries. So soon as these are distended with the blood column "congestion" is present.

The next stage is "exudation" of the serum into the surrounding areolar spaces, and is the immediate antecedent of perceptible "swelling."

When the swelling impinges upon the sensory nerve tracts so as to minify the thread of neurine to the degree of obstructing the afferent current, "pain" is present, completing the essential conditions of the inflammatory process. "Redness" may or may not be present, but usually is. With proper management "resolution" may yet be invoked without the formation of "pus" by *death* of *blood, fluid* and *corpuscular*, within and without the congested territory. Should resolution not take place, the inflamed territory will augment until exercise of the part is inhibited, from debility, or from intensity of the pain; "debility" from paralysis of the motory system, and "pain" from obstruction of the sensory system of nerves. As soon as the plus quantity of radiancy, which polarizes the corpuscles and renders the serum plastic, is exhausted, death asserts its dominion by inaugurating chemical change through deoxidation and production of ptomaines so destructive to integrity of the part by their alkaloidal and poisonous character, perverting or arresting physiological activity.

When the oxy-hæmoglobin is deprived of its loosely-held oxygen, it is reduced to inert hæmoglobin, and there being no free oxygen present to repolarize it by converting the now plain oxide of iron into a sesquioxide by appropriation of a half equivalent of the oxygen, the globule readily melts into "*sanies.*"

The next product in the retrogressive chemical change is this: Hæmatin is diffused throughout the broken-down corpuscles and serum constituting the sanies, which now takes hold of nerve and muscle, killing and destroying them, and dissolving them into a product termed "ichor," which now in turn attacks sinew and bone, producing fearfully destructive ptomaines, against which not even white and yellow connective tissue corpuscles are able to stand, though these be the last to yield to the solving process of alkaloidal power, resulting in "phagedena" and "gangrene." The principles of cure consist in destroying poisons, and removing dead and dying tissue by mechanical, chemical or dynamic means.

TUESDAY EVENING JAN. 18TH.

The society again convened at the Masonic Temple; over six hundred gentlemen were present, completely filling the large hall.

Dr. N. W. Kingsley read a paper, entitled "Critical Essay on Treatment of Irregularities." He produced maps or sketches illustrating a number of regulating cases, with appliances in position and teeth being operated upon. He stated there could be no positive system in tooth-regulation. He had invented many appliances for various cases presented, and seldom employed the same arrangement for a number of cases.

In treating irregularities we had nothing to do with anatomy, physiology or therapeutics, but should be governed by the best mechanical laws. Referring to one of his diagrams, he called attention to the so-called Coffin appliances, and to Dr. Coffin's statement at the International European Congress, that he had treated 2,500 cases with his piano-wire springs and vulcanite plates. Dr. Kingsley had also tried them, but found that too much time was required to accomplish the desired results with them, and obtained the same ends by other means and in less time.

Figures were also exhibited, showing piano-wire appliances of Drs. Newkirk, Talbot and Caldwell. In criticising these cases the essayist stated that where the back teeth articulated well and the front teeth were much mixed up, it was often better to do some extracting than to put in an elaborate appliance to correct the trouble, and he believed the methods he adopted were the most simple and easiest for patients. He also had diagrams representing cases treated by other dentists.

Dr. Talbot, of Chicago, in response to an interrogation, explained how he produced certain results, which Dr. Kingsley did not appear quite to understand. Dr. Talbot carefully pointed out the several steps taken, giving his reasons for the same. He also stated that Dr. Coffin, of London, was greatly to be commended for his piano-wire and vulcanite plate combination, and that he (Dr. T.) had obtained very good results by using them.

Representations of cases of Dr. Keely, of Ohio, and Dr. Farrar, of New York, were shown by Dr. Kingsley. Case 18, of Dr. Farrar, was a complicated one, consisting of a centre piece with a line of jack-screws and other arrangements in profuse number. Dr. K. could see no necessity for such complicated affairs.

Dr. Farrar described the appliance alluded to, stating that it worked nicely, and that the patient soon became accustomed to it.

He takes pleasure in studying out and constructing such appliances, as he is very fond of mechanics.

Dr. Kingsley considers that for the treatment of irregularities the simplest and easiest methods are best.

Dr. J. Rollo Knapp, of New Orleans, read a paper on "Crown and Bridge-work," which will be found in *The Cosmos* for February.

Dr. Frank French, of Rochester, read an essay entitled "What Caused It?" He cited a case where, in the mouth of a lady, was an inferior molar with fillings of both gold and amalgam. She complained of a pronounced metallic taste and a profuse flow of saliva; on the same side of the face the gland was much enlarged. The face was bandaged with a linen compress, and although no medicinal application was made, the surface of the linen against the skin had a reddish-brown color when removed. Some of the patient's friends imagined it a case of mercurial poisoning, so the amalgam filling was removed and a gutta percha stopping substituted. The saliva was tested and found normal. Query—What gave the color to the compress?

THE CLINICS.

These were of the greatest interest to the visiting dentists, and many new methods and implements were exhibited. They were given at the rooms of the New York College of Dentistry, commencing Tuesday, January 18th. It is estimated that at least six hundred dentists were in attendance during the day.

Dr. Olga Neyman, of New York, filled a left upper lateral, the cavity being in the mesial surface, using Quarter Century foil, which was introduced by the hand mallet.

Dr. Sophie E. Feltwell, of Pittsburgh, Pa., placed a gold crown upon a right lower second bicuspid in the mouth of a patient for whom Dr. Kirk, of Philadelphia, Pa., had implanted a left upper central incisor, which appeared to be satisfactory in every respect.

Dr. Genese, of Baltimore, exhibited a very ingenious dental speculum and mouth gag combined, which was also a lip holder and tongue compressor.

Dr. T. W. Brophy, of Chicago, filled a right lower first molar with gold, demonstrating the use of his new loop matrices. The cavity was situated in the distal and grinding surface of the tooth.

Dr. Madden, of the Empire City Electric Co., exhibited a new battery with only two cells, and a new electric dental motor.

The S. S. White Dental Manufacturing Co. had their great variety of dental instruments and teeth on exhibition, making a really wonderful display, and gave to visitors a more comprehensive idea of the resources of dentistry than could be obtained in any other way.

Dr. D. A. Williams, superintendent of the mechanical department of the New York College of Dentistry, exhibited five patients with fractures of the lower jaw, wearing interdental splints, which can be constructed from ordinary red gutta percha in about one hour.

Dr. W. Irving Thayer, of Brooklyn, filled a right upper lateral in the distal surface with E. Kearsing's gold, using for the introduction his new trip hammer. He also made some experiments out of the mouth.

Dr. J. A. Steurer, of New York, made some experiments with his new plastic gold.

Dr. Geo. F. Reese, of Brooklyn, cast a partial lower plate of his metal.

Dr. Henry W. Morgan, of Nashville, exhibited two upper central incisors, the roots of which were very short, and bent at right angles ; also the roots of two other upper central incisors, which were exceedingly short.

Dr. Wm. Crenshaw, of Atlanta, Ga., filled for Dr. E. P. Brown a right upper first molar, the cavity, which was extensive, involving the mesial and grinding surfaces. He used for the introduction of the gold the electro-magnetic mallet.

Dr. T. D. Shumway, of Plymouth, Mass., filled a right upper lateral in the distal surface, using in the commencement of the operation White's velvet cylinders, followed by Nos. 3 and 4 of 1,000 fine foil, introduced by his ivory points.

Dr. R. J. Verplank, of Albany, N. Y., exhibited a new separator and matrix combined, made of two pieces of watch spring attached to two jack screws.

Dr. W. W. Evans, of Washington, D. C., filled a right lower second bicuspid, the cavity occupying the cervico-buccal portion of the tooth. He applied his new adjustable clamp for holding the rubber dam above the cavity, using No. 4 Rowan's gold, which was introduced by his mechanical mallet.

Dr. S. G. Perry, of New York, filled a left lower first molar in the mesial and grinding surfaces with gold, at the same time demonstrating the use of his separators. He used his new engine, which attracted great attention, and by many was declared to be the best engine ever produced.

Dr. J. G. Morey demonstrated the use of his nerve and crown drills.

Dr. John A. Daly, of Washington, D. C., exhibited numerous specimens of sets of teeth on rubber which had been lined with his gold.

Prof. Frank Abbott, of New York, exhibited his new automatic plugger with back action, which attracted much interest.

Prof. S. H. Guilford, of Philadelphia, Pa., exhibited his new set of steel matrices; also a new galvanic battery for the electro-magnetic mallet, which was afterward used by Dr. F. D. Gardiner.

Mr. W. H. Baldwin, a student of the New York College of Dentistry, exhibited a new mechanical mallet.

Dr. Geo. F. Reese, of Brooklyn, inserted four central incisor and a left upper first bicuspid crown, the posts of which had been cast in his metal.

Dr. C. E. Francis, of New York, exhibited the new A. P. Merrill gum forcep, and demonstrated its use in the mouth.

Dr. H. C. Register, of Philadelphia, exhibited his new engine and hot air and spray apparatus.

The Gibbs Electric Co., of New York, exhibited their electric motor and a mouth illuminating apparatus.

Dr. J. P. Geran, of Brooklyn, filled a right upper second bicuspid in the grinding surface with R. S. Williams' gold, which was introduced by the Herbst method.

Dr. G. W. Melotte, of Ithaca, made for Dr. L. S. Straw, of Newburgh, N. Y., a piece of bridge-work with two teeth. The bridge was attached to a cap enclosing the right upper second molar in the back, bridging over the first bicuspid, which was loose, the whole secured in front by means of a collar applied around the cuspid.

Dr. Lawrence Vanderpant, of Orange, N. J., showed a lower cheoplastic plate in combination with pink rubber, which had been exhibited at a meeting of the Odontological Society of Great Britain in 1866; also four other lower sets of the same material, in which C. Ash & Son's diatoric teeth had been employed. He also exhibited a variety of artificial crowns made from C. Ash & Son's tube teeth.

Dr. B. H. Teague, of Aiken, S. C., sent a very serviceable arm rest, to be used at the chair during long operations, and some of his depressed disks, which were distributed.

Dr. Parr demonstrated his quick method of making and setting artificial crowns, the tooth being a left upper lateral. The root was first cut off, the labial edge being below the gum, the lingual left considerably longer. The root was then cut round with a disk, and a strap of 22 carat gold of 32 gauge quickly fitted around it with pliers. The edge of the strap was then cut even with the root surface, and a pure gold floor was fitted on it, being pushed up generally to fit the root surface. The strap and floor were then soldered together, and a hole drilled in the latter to receive the pivot, a three-cornered piece of platino-iridium wire. A plate tooth was then ground to fit the cap, backed by burnishing upon it a plate of the same pure gold that formed the floor, and the three parts—cap, pivot, and crown—were then joined with hard wax, put together in place on the root, removed, soldered, and finished. The completed tooth was set with oxy-phosphate. The doctor worked under great disadvantages, lacking many necessary facilities, and experiencing many annoying interruptions, in spite of which the operation was finished in one and one-quarter hours.

Dr. J. Rollo Knapp, of New Orleans, La., demonstrated his method of crown and bridge-work, which, as usual, was one of the great attractions of the Clinic.

CLINIC GIVEN WEDNESDAY, JANUARY 19TH.

Dr. C. F. W. Bödecker demonstrated the preparation of microscopical specimens, at the same time explaining several methods of treating tissues previous to cutting. To soften teeth and bone he advised the treatment with a solution of chromic acid of the strength of a half to one per cent. In about a quart of this solution a small number of teeth are immersed, renewing the solution at least once every week, and adding from two to five drops of dilute hydrochloric acid. This method may be employed for adult teeth, while in position in the jaws, as well as for embryos. Many other methods are employed, but in order to avoid confusion Dr. B. stated that he would only mention the simplest and most efficient. The treatment with chromic acid has for its object the softening of the hard tissues, and the hardening of the soft ones, such as muscles, skin and myxomatous tissues.

When the tissues to be cut are of the proper consistence, which for teeth requires from two to three months' preparation, they must be imbedded either in paraffine and wax, or in celloidine. Previous to imbedding, the tissues must be laid in absolute alcohol for twenty-four hours, to abstract the water which they have absorbed from the chromic acid solution. They are then immersed in the celloidine, which is dissolved in sulphuric ether, and the latter allowed to evaporate slowly. After twenty-four hours the vial containing the celloidine is filled with absolute alcohol, which further hardens this substance, and after another lapse of a day will make the celloidine about the consistency of hard butter, when the preparation is ready for cutting. If this is done by a microtome, the imbedded tissue must be fastened to a cork, to which a piece of paper has been attached by means of a rather thick solution of gum. When the paper has been secured and well dried, a rather thin solution of celloidine is poured upon it, and the imbedded tissue put in the desired position upon the paper, and after five or ten minutes the cork, with the tissue to be cut, is immersed in alcohol again for about one day, when it is ready for cutting. During the process of cutting the tissue must be kept wet with absolute alcohol by means of a drop tube.

Dr. Bödecker explained that it was impossible to take a single specimen, soften, imbed, cut and mount it at one clinic. Therefore, after he had demonstrated the imbedding of specimens, he took another preparation from which he had previously cut some sections, and demonstrated the cutting by means of a microtome. When several good sections had been obtained, they were immersed in water, to which a few drops of carmine* had been added, and as there was no time to wait for staining the sections perfectly, which usually requires from six to twenty-four hours' time, they were removed from the staining fluid and mounted at once. The sections, which were from the lower jaw of a six to seven-months' human fœtus, were transported to the glass slide by means of a copper spoon, a little glycerine diluted with water added, and the specimen covered with a thin square glass cover. The surplus glycerine was

*The carmine solution is prepared by adding one drachm of carmine to one ounce of distilled water, with ten drops of aqua ammonia, and then filtering.

then very carefully cleaned off from the slide all around the cover by means of bibulous paper, and the latter was cemented to the slide by painting a rim of ordinary black asphalt paint around the edge of the cover. The specimen was exhibited under the microscope, and examined by most of the gentlemen, while Dr. B. explained the different parts of the fœtal tooth which were in the field of the microscope.

Dr. C. A. Timme, of Hoboken, N. J., filled a right upper first molar, the cavity of which was very large, occupying the mesial and grinding surface, using Wolrab's gold, which was introduced by the Herbst method. He also exhibited a new hypodermic syringe, to be used with the cocaine or morphia tablets.

Dr. S. G. Perry, of New York, filled a right upper first molar in the grinding surface with No. 4 Globe gold foil, which was introduced by means of glass rods, the points of which had been drawn out, curved at various angles, and rounded off in the flame of a Bunsen burner. These glass instruments were used in about the same manner as Dr. Abbott's hand condensors (see INDEPENDENT PRACTITIONER for February, page 81). The gold was condensed with these points to the satisfaction of all who examined the completed filling.

Dr. M. L. Rhein, of New York, filled for Miss J. a right superior first molar. The patient was seen for the first time on January 15th, when the tooth was found with a large amalgam filling occupying the anterior approximate, crown, and a large portion of the palatine and buccal surfaces. The filling was loose, and only held in position by the adjacent bicuspid. On removal a putrescent pulp was found, which was thoroughly removed by the aid of the Morse drills, and the cavity was disinfected with a bichloride of mercury solution, the root being filled with gutta percha and oxyphosphate of zinc at the same sitting. The tooth was perfectly comfortable at all times following. It was contoured at the clinic of January 19th, with gold, using a quarter ounce of the Wolrab German gold. The operation was performed by a combination of the Herbst rotary method and the electric mallet, the two methods being used alternately during the operation.

Dr. E. S. Talbot, of Chicago, exhibited his coiled wire spring for regulating teeth, in connection with many models, to demonstrate the various situations in which it can be applied.

By the use of gold or platinum bands around the teeth, or by using rubber plates, drilling retaining pits in sound tissue is avoided. This spring has several advantages over the Coffin plate. It requires less care in adjusting. With the Coffin plate it is uncertain which end has the greatest force, because it is distributed through a small body attached to a large one, which must exert its influence on the body to be moved. With this spring the force is exerted between two points, regardless of the shape of the wire or manner of application.

Another point in favor of this mode of operating is, that in mechanics it works better to have the force distributed from a small space to the body to be moved, rather than to have the end of the wire attached to a broad surface through which the force is distributed to the body to be moved. In the models exhibited, the appliances could, with one or two exceptions, be removed and cleansed by the patient without necessitating the attention of the dentist oftener than twice a week, and in one case the patient attended to adjustments for six weeks without any attention whatever. The advantage of the coil over a straight wire is exemplified in Dr. Coffin's W-shaped spring. He recognizes the advantage of a complexity of wires to obtain a uniform pressure. The Helix spring, in mechanics, is acknowleged to be the simplest form of spring, and is used in all small machinery, such as sewing machines, reed organs and watches, etc. From one to three coils will produce a gentle, uniform and constant pressure, at any required distance. The pressure can be regulated by changing the size of the wire. When the patient is young, and the teeth move easily, No. 18 will serve the purpose. From Nos. 18 to 23 may be used in ordinary cases, and No. 24 when the patient is older and the bones are dense. The object, however, is not to do the regulating rapidly, but slowly and surely.

In making the spring we drive a needle or wire into the bench, and with a hand vise catch it where the coil is to begin, and wind this about the needle as many times as it is to be coiled. Stop at the point it started from, that the coil may be inside the spring. This will give the arms of the spring a long range. Then cut the wire about two inches from the coil, which will give ample length for regulating any of the teeth. This can be cut to suit the case.

The wire will oxidize in the mouth, but retains a smooth surface, and will not be injurious, as it does not rust.

Dr. E. P. Brown, of Flushing, L. I., inserted for Dr. Wm. Crenshaw, of Atlanta, Ga., a right upper second bicuspid all porcelain bridge, which was anchored in the distal surface of the first bicuspid and the mesial surface of the first molar. In both of these teeth when the bridge was inserted, there were large cavities which were filled with gold, introduced by means of the electro-magnetic mallet. This operation attracted much attention, and was highly commended by those who witnessed it.

Dr. Chas. Hubbard, of New York, exhibited an upper bicuspid tooth which, in the year 1881, had been extracted, filled with gold, and replanted at the same sitting, by Dr. White. The tooth came under the observation of Dr. Hubbard in January, 1883, when it appeared to be perfectly healthy, except that the gum was slightly inflamed, and it had given the patient no trouble whatever, except for the first two days after it had been replaced. In October, 1884, the patient brought the tooth to Dr. Hubbard, stating that it had fallen out of its own accord. The root of the tooth was absorbed to such an extent that the remains of it, which measured only about one-quarter of an inch in length, in shape resembled a knife blade.

Dr. C. C. Carroll, of Meadville, Pa., demonstrated the use of aluminum in prosthetic dentistry.

This clinic clearly proved the practicability of making a complete dental plate of aluminum by casting, and the possession of the desired properties of great lightness, strength and durability.

The appliances used in making the cast are very simple, and apparently easy of employment, consisting of a cylindrical crucible, with a closely fitting tubular stopper, to which was attached a rubber bulb, by means of which the melted aluminum was injected into the matrix invested in an iron flask, with nipple attachment connecting with the crucible. Both the crucible and flask were heated in a small double chamber gas furnace, constructed to act automatically, so that when the aluminum was melted in the crucible placed in the lower chamber, the flask containing the matrix and invested teeth, placed in the upper chamber, will be raised to a temperature somewhat below the melting point of aluminum, and thus prevent the checking of the teeth in the act of casting. When the aluminum was melted, which required about six minutes, the crucible was placed above and in connection with the flask, and the molten aluminum forced by air into all parts of the matrix, producing a very perfect dental plate.

The difficulties formerly experienced in some mouths, by the etching of aluminum plates into holes, were explained by Dr. Carroll as being due to iron and other impurities contained in the aluminum used, and that the aluminum of commerce is entirely unsuited for dentures, on account of these impurities and its great tendency to contraction, which difficulties and objections are entirely overcome in the aluminum as prepared by Dr. Carroll.

He exhibited an aluminum solder, and demonstrated its use by soldering a hole made in an aluminum plate, using a common copper soldering iron without investing the plate, thus accomplishing very easily what has been regarded heretofore by workers in this metal a thing very difficult or impossible to be done.

This clinic elicited marked interest and much commendation on the part of all who witnessed it.

The teeth used were the S. S. White's ordinary rubber teeth, and the method of mounting was that usually employed for rubber work, except in the use of a very thin paraffine base plate of about No. 23, gold plate gauge.

Dr. R. F. Hunt, of Washington, D. C., exhibited specimens of vulcanite work lined with gold.

Dr. G. W. Melotte inserted the bridge piece made the day previous for Dr. L. S. Straw.

Dr. Register, of Philadelphia, Pa., exhibited a practical working pneumatic pump as a motor for his pneumatic mallet.

Dr. J. N. Farrar, of New York, exhibited illustrations of his method of regulating teeth.

Dr. Frank D. Gardiner, of Philadelphia, Pa., filled an upper molar; the cavity occupied the mesial and grinding surfaces. The gold was introduced by means of the electro-magnetic mallet, using a battery which had been exhibited the day previous by Prof. S. H. Guilford.

Dr. Wm. H. Atkinson, of New York, presented a patient for whom he had implanted two inferior incisors some months ago.

Dr. J. M. Crowell, of New York, carved a set of block teeth, which were baked and enameled.

Dr. W. P. Horton, Sr., of Cleveland, Ohio, exhibited Dr. Husted's pneumatic mallet, which was worked by a pump attached to a dental engine.

Dr. William H. Mitchell, of Bergen Point, N. J., exhibited an engine attachment for automatic mallets and pluggers.

The device, when attached to the instrument, does not interfere with the automatic blow, but enables the operator to use the rapid blow given by the device connected with the hand-piece of the engine, or the single stroke of the automatic at pleasure, without being detached from the hand-piece.

The device can be applied to all automatic mallets, but the one on exhibition was applied to the Richmond Automatic Plugger.

The blow given is rapid, and the sensation similar to that of the electric mallet.

Dr. John H. Meyer, of New York, made an upper continuous gum set, from the impression which was taken on the day previous. The set which had been carved, baked and enameled at the clinic, was satisfactory in every respect.

Dr. H. A. Parr, of New York, made and inserted a porcelain faced crown for an upper canine in the same manner as on the day previous.

Dr. J. Mapp filled a right upper third molar in the mesial and grinding surfaces.

Dr. R. W. Starr, of Philadelphia, demonstrated the removal of a live pulp with engine drills, applying an obtunder which, however, was unsuccessful.

THE BANQUET.

The banquet at Mazetti's was a fitting *finale* to this most interesting anniversary gathering. The menu was a very choice one, consisting of thirteen different courses, with eight varieties of wine. Plates were laid for 165 persons, the full capacity of the tables.

The first toast of the evening, as printed upon the menu, was the following quotation from Shakespeare: "Bid these gentlemen welcome; come! we have a hot venison pasty to dinner; come, gentlemen, I hope we will drink down all unkindness."

Dr. Wm. Carr, the President of the society, gave a brief welcome, and then introduced Dr. J. W. White, of Philadelphia, to respond to the toast, "Our Invited Guests." He began by telling, in his inimitable manner, a number of funny stories, and then said:

"What shall we, the invited guests, say of our hosts, the First District Dental Society? What, but that it has given the best and greatest meeting of our profession that has taken place since the world began. And the influence of this meeting; what shall it be? It will last until time's last whirlwind sweeps the sky."

Dr. C. A. Marvin, of Brooklyn, responded to the toast, "The Dental Profession." He said he was profoundly impressed with the wisdom of the officers of this society, for they had gathered together dentists from all sections of the United States. Never was a time in the history of dentistry when there was a greater number of dignified and able men engaged in its practice in the United States than now. The cause of extra growth of dentistry is due to our Associations.

The Rev. Dr. Brady E. Backus responded very felicitously to the toast "The Pulpit;" telling a number of laughable stories, and closing with a defense of the pulpit from the charge that its power is waning.

The next toast, "The Dental Society of the State of New York," was responded to by Dr. N. W. Kingsley, the President of the society. This was not one of the regular toasts as announced upon the menu, but was introduced at the request of Dr K., that he might be given an opportunity to speak concerning the coming Medical Congress. After some preliminary words concerning what of late has been his custom in making after-dinner speeches, he said that in speaking for the society which he represented to-night, he was individually responsible for what he should say. There was upon the bill of fare, as before them, one dish that had not been served others, although it was then in his mouth. He had gained considerable notoriety, he would not say reputation, of late, by opposing the dental section of the proposed Medical Congress, and arguing that dentistry was not a specialty of medicine. He proceeded to admit that "dentistry is a part of the healing art" and to regret that he had opposed the dental section of the Medical Congress, and hoped that all who were present at this, the grandest assemblage of dentists since the world began, would work earnestly to make the section a grand success.

Dr. W. W. Allport, President of the American Dental Association, then responded briefly to a toast in honor of that society, and at the midnight hour the meeting adjourned.

